# Dietary modulation of gut microbiota and its role in atopic dermatitis: integrative evidence from animal and human studies

**DOI:** 10.3389/fimmu.2025.1635262

**Published:** 2025-10-09

**Authors:** Fang Xu, Xinyue Jiang, Yuyang Jin, Yadi Yang, Xinyue Chen, Ying Chen

**Affiliations:** ^1^ School of Public Health, Kunming Medical University, Kunming, Yunnan, China; ^2^ Yunnan Maternal and Child Health Hospital, Kunming, Yunnan, China; ^3^ Yunnan Provincial Key Laboratory of Public Health and Biosafety & School of Public Health, Kunming Medical University, Kunming, Yunnan, China; ^4^ Yunnan Key Laboratory of Cross-Border Infectious Disease Prevention and New Drug Development, Kunming, Yunnan, China

**Keywords:** atopic dermatitis, gut microbiota, dietary factors, 16S rRNA high-throughput sequencing, microbiota functional prediction

## Abstract

**Background:**

Atopic dermatitis (AD) is associated with disturbance in the gut microbiota, but the dietary factors behind this dysbiosis are still unknown. Therefore, we investigated how food choice patterns impact the gut microbiota, which in turn influences the development and progression of AD.

**Methods:**

A mice AD model washed using 2,4-dinitrochlorobenzene (DNCB). After 4 weeks, epidermal histopathology, serum immunoglobulin E (IgE) levels, and gut microbiota profiles were assessed. At the same time, we recruited 102 clinically diagnosed AD patients and 102 age- and sex-matched controls. Participants completed a food frequency questionnaire and provided stool samples to analyze dietary patterns, gut microbiota diversity, composition, function, and their associations.

**Results:**

In mice, AD induction caused marked epidermal thickening, inflammatory infiltration, and a dose-dependent increase in serum IgE (up to ~3.0-fold compared to control, p < 0.01). Alpha diversity analysis revealed a significantly higher ACE index in the high-dose group (p < 0.05), whereas the Chao, Shannon, and Simpson indices did not exhibit significant changes. In humans, microbial diversity declined markedly (Shannon index, −20%, p < 0.001), with reductions in *Firmicutes* and *Bacteroidota*, but enrichment in *Actinobacteriota* and *Bifidobacterium*. Dietary patterns in AD patients showed lower consumption of refined grains (-24 g/day) and higher intake of vegetables and fruits (+38 g/day), which strongly correlated with microbial shifts. Functional predictions revealed reduced carbohydrate, amino acid, and energy metabolism pathways. Together, these findings suggest a novel diet–microbiota–immune axis in the pathogenesis of AD.

**Conclusions:**

Evidence from mice to humans suggests that reduced intake of refined grains and increased consumption of plant-based foods are associated with remodeling of the gut ecosystem – including reduced diversity and metabolic capacity – which may play a role in AD. These findings are exploratory and should be considered hypothesis-generating, warranting validation in prospective studies. These findings provide a theoretical and scientific basis for future research on dietary interventions and gut microbiota modulation strategies for preventing and treating AD.

## 1 Introduction

Atopic dermatitis (AD) is a common chronic inflammatory skin disease characterized by recurrent episodes of intense itching, eczematous lesions, and compromised skin barrier function (1, 2). Recently, the global prevalence of AD has significantly increased, imposing substantial economic and psychosocial burdens on patients and their families (3) Although genetic predisposition, environmental exposures, immune dysregulation, and impaired skin barrier function have been strongly associated with AD pathogenesis (4, 5), its precise pathophysiological mechanisms remain incompletely understood, and existing treatment approaches demonstrate limited efficacy.

Accumulating evidence underscores the pivotal role of the gut microbiota in the pathogenesis of atopic dermatitis (AD) (6, 7). Microbial dysbiosis can lead to increased intestinal permeability and immune dysregulation, thereby promoting chronic inflammatory responses (8, 9). However, studies specifically addressing how dietary factors shape the composition and metabolic functions of gut microbiota in AD patients are still limited. In particular, there is limited understanding of the effects of different types of food intake or components on gut microbial diversity and function in AD populations. Most existing research has relied on cross-sectional human studies or isolated animal experiments, lacking integrative approaches that combine animal models with human data. This gap limits our understanding of the causal relationships among diet, gut microbiota, and AD. Given that diet exerts a profound influence on the gut microbial ecosystem—unhealthy patterns, such as high-sugar and high-fat diets, promote dysbiosis, while fiber-rich diets increase beneficial bacteria and support gut homeostasis (10, 11) — there is a clear need for comprehensive investigations into the specific dietary determinants of microbiota changes in AD.

Recent studies have further demonstrated that habitual dietary patterns significantly influence AD risk and severity. For example, high estimated total fat intake has been associated with enhanced allergy sensitization and increased prevalence of atopic diseases in young adults (12). Conversely, frequent consumption of plant-based foods has been shown to reduce the risk of AD exacerbation (13). Other findings suggest that a high intake of trans and saturated fatty acids may increase susceptibility to AD flare-ups (14). In contrast, diets rich in fiber and probiotics are associated with a lower risk of AD and reduced house dust mite allergy (15). These findings support the hypothesis that dietary modulation can shape immune responses and gut microbial dynamics, aligning with the current study’s objective to elucidate the diet–microbiota–AD axis.

Based on the above research gaps, this study adopted a combination of animal experiments and population studies to systematically analyze the effects of dietary factors on the diversity, composition, and function of intestinal microbiota, and further comprehensively explore their relationship with the pathogenesis of AD. Specifically, we first established a DNCB-induced mouse model of AD to investigate changes in skin histopathology, serum IgE levels, and the typical gut microbiota composition. Subsequently, we expanded our exploration to the human population, examining the diversity, composition, and function of the gut microbiota in AD patients, and further investigating the correlation between different dietary patterns and microbiota composition. We hypothesize that specific nutritional patterns can causally alter gut microbiota composition and functional capacity, which in turn modulate immune responses and influence the risk and severity of AD. This design allows us to investigate both the mechanistic pathways in a controlled animal model and the corresponding associations in human populations, thereby providing an essential theoretical basis and scientific evidence for dietary intervention and microbiota modulation strategies in the prevention and treatment of AD.

## 2 Materials and methods

### 2.1 Animal experiments

#### 2.1.1 Establishment of the AD mouse model

In this study, male BALB/c mice aged 6–8 weeks (corresponding approximately to young adulthood in humans) were randomly assigned to three groups (n = 6 per group): a control group (matrix solvent, acetone: olive oil=3:1), a low-dose DNCB group (low-concentration DNCB, 0.1%), and a high-dose DNCB group (high-concentration DNCB, 0.5%), with six mice in each group. After 7 days of acclimatization of all mice under controlled temperature, humidity, and pathogen-free conditions, an area of 1×1 cm² on the dorsal skin of each mouse was shaved, and a mouse AD model was induced by DNCB (exposure dose 200 μg/time). Detailed sensitization procedures were described in **Table 1** (16).

**Table 1:** Sensitization protocol for DNCB-Induced atopic dermatitis (AD) in mice.

Group	Week 1	Frequency	Weeks 2–6	Frequency
Control	Vehicle	2 times/week, every 3 days	Vehicle	2 times/week every 3 days
Low-dose	1% DNCB	2 times/week every 3 days	0.1% DNCB	2 times/week every 3 days
High-dose	1% DNCB	2 times/week every 3 days	0.5% DNCB	2 times/week every 3 days

#### 2.1.2 Histopathological evaluation of mice skin

At the end of the experiment, mice were euthanized by cervical dislocation. Dorsal lesional skin was excised and fixed in 4% paraformaldehyde, paraffin-embedded, sectioned at four μm, and stained with hematoxylin and eosin. Epidermal thickness, keratinization, necrotic cell debris, and inflammatory infiltrates were examined under a light microscope.

#### 2.1.3 Serum IgE measurement

After the experiment, blood was collected from the retro-orbital plexus. Serum was isolated by centrifugation (3000 rpm, 10 min, 4 °C), and total IgE concentrations were quantified using a commercial ELISA kit (Jincheng Institute of Bioengineering, Nanjing, China.), following the manufacturer’s instructions. Results were expressed as ng mL^−^¹.

#### 2.1.4 Fecal sample collection

After final treatment, fresh fecal pellets (~100 mg) were aseptically collected directly from the rectum of each mouse, ensuring that there was no additional contamination. Samples were immediately placed in pre-chilled sterile tubes, snap-frozen in liquid nitrogen, and transferred to a -80 °C freezer within 10 minutes for subsequent DNA extraction and 16S rRNA sequencing analysis.

### 2.2 Human study

#### 2.2.1 Sample size calculation

Based on the prevalence of AD (11%) (17), the sample size required for the population study was calculated using a sample size estimation formula to ensure the scientificity and representativeness of the study, and 204 participants were recruited: 102 clinically diagnosed AD patients and 102 age- and sex-matched healthy controls. All subjects completed a structured questionnaire (diet frequency and demographics) and provided stool samples. The required sample size was calculated according to the formula for unmatched case–control studies (18, 19):


$$n=\frac{{\left(Z_{\alpha /2}＋Z_{\beta }\right)}^{2}\times [p_{0}(1-p_{0})+p_{1}(1-p_{1})]}{p_{1}-p_{0}^{2}}$$

where $Z_{\alpha /2}$ is the standard normal deviate for the two-tailed significance level (α = 0.05, $Z_{\alpha /2}$ = 1.96), $Z_{\beta }$ is the standard normal deviate for 80% power, $p_{0}$ is the expected proportion of low gut microbiota diversity in controls, and $p_{1}$ is the anticipated proportion of cases. Based on previous studies of microbial diversity in atopic dermatitis populations, we assumed $p_{0}$ = 0.30 and $p_{1}$ = 0.50, corresponding to an odds ratio of 2.33. These assumptions were based on effect sizes reported in prior microbiome case–control studies of AD (20). While such values allowed us to achieve >80% power with 102 participants per group, we acknowledge that microbiome data are inherently variable, and actual effect sizes may differ across populations. Therefore, the present sample size should be regarded as sufficient for exploratory analysis, but larger cohorts will be required to confirm the stability of these associations. Substituting these values yielded a minimum of 93 participants per group. To account for potential non-response and sequencing failures (~10%), the final sample size was set at 102 per group.

#### 2.2.2 Recruitment, inclusion, and exclusion criteria

Participants were randomly selected from the dermatology outpatient clinic.

Inclusion criteria, AD group:

1. Symmetrical eczematous lesions persisting > 6 months;
2. At least two laboratory indicators: elevated peripheral eosinophil count/percentage, increased total serum IgE, and allergen-specific IgE ≥ Class 2;
3. Moderate-to-severe cases may present with elevated serum lactate dehydrogenase (~30%).

Inclusion criteria, control group:

Outpatients without AD or other inflammatory skin disorders.

Exclusion criteria: Diarrhoea, diabetes, ulcerative colitis, Crohn’s disease, or other active infections; recent chemotherapy, radiotherapy, or major surgery; use of antibiotics, corticosteroids, herbal medicines, or probiotics within 3–6 months (regular long-term probiotic use permitted); marked dietary changes within 1 week; sampling deferred during menstruation.

#### 2.2.3 Food-frequency questionnaire

After obtaining informed consent, each participant completed an interviewer-administered FFQ via the Wenjuanxing online platform, which covered dietary intake over the preceding 12 months. A semi-quantitative food frequency questionnaire (FFQ) was used to assess participants’ habitual dietary intake. This FFQ was adapted and shortened from the validated questionnaire applied in the China National Nutrition and Health Survey (CNNHS) and the China Health and Nutrition Survey (CHNS), which have been widely used in large-scale epidemiological studies of Chinese populations. The adapted version retained major food groups relevant to dietary assessment of atopic dermatitis, including staple foods, vegetables, fruits, meats, dairy products, and snacks. Although the modified FFQ was not independently re-validated, it was derived from established instruments with proven reproducibility and validity in Chinese adults. Foods were classified into seven categories:

1. Refined grains (rice, fried dough, instant noodles)
2. Whole grains (whole-wheat bread, buckwheat, maize, millet, potato, sweet potato)
3. Vegetables (root/gourd; leafy greens; cruciferous; fresh legumes)
4. Fruits (fresh, dried, candied; 100% juice)
5. Dairy products (milk, yoghurt, processed dairy)
6. Protein sources (poultry/red meat, processed meat, fish/seafood, eggs, offal)
7. Snacks and sweets (high-sugar/fat snacks, nuts, fried snacks).

Dietary data from the FFQ were categorized into predefined food groups based on nutritional composition and prior literature (Chinese Dietary Guidelines, 2022; USDA Food Grouping System). Specifically, staple foods (e.g., rice, wheat-based products) were grouped as cereals, while vegetables, fruits, dairy, meats, oils/fats, snacks/sweets, and beverages were treated as separate categories. Butter and margarine were classified under the “fats and oils” group, rather than “dairy products,” due to their high fat content and culinary use. Similarly, ice cream and chocolate were categorized under “snacks/sweets” rather than “dairy.” High-fat foods (e.g., fried snacks, fatty meats, butter, margarine) were grouped according to food type but also considered separately in sensitivity analyses, with high-fat classification defined as ≥20 g of fat per 100 g of food. This standardized categorization ensured consistency and reproducibility in dietary pattern analysis.

The FFQ used in this study did not separately assess foods or beverages containing probiotics (e.g., yogurt, cultured milk drinks). Instead, these items were subsumed under the broader ‘dairy products’ category. We acknowledge that this may reduce sensitivity in capturing diet–microbiota associations driven specifically by probiotic foods.

#### 2.2.4 Fecal sample collection

Participants collected morning stool samples in sterile tubes, ensuring they were free from urine or water contamination. Samples were kept on ice and delivered to the laboratory within two hours, then stored at -80 °C for DNA extraction and microbiome analysis.

### 2.3 16S rRNA high-throughput sequencing

Genomic DNA from mouse and human feces was extracted with a commercial kit. The V3–V4 region of the 16S rRNA gene was amplified using primers 341F/806R, purified, and sequenced on an Illumina MiSeq platform (2 × 300 bp). Raw reads underwent quality control, denoising, and OTU clustering (with a 97% similarity threshold) in QIIME2. Taxonomic assignment, alpha/beta diversity, and functional prediction (PICRUSt2) were then performed.

### 2.4 Statistical analysis

Data were analyzed with SPSS 26.0. Normally distributed variables were compared by one-way ANOVA; non-normal data by Kruskal–Wallis H test. *Post-hoc* comparisons were performed using the Bonferroni or Mann–Whitney U tests. Spearman’s rank correlation was used to assess associations; P < 0.05 was considered statistically significant. Microbiome data were processed through the i-Sanger online QIIME2 pipeline.

## 3 Results

### 3.1 Animal experiment

#### 3.1.1 Skin pathology and serum IgE levels

**Figure 1:**
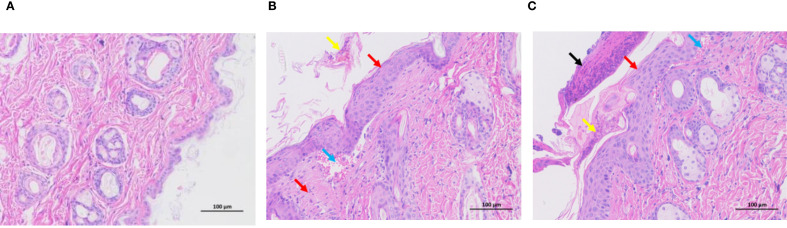
Representative histopathological changes of dorsal skin in mice (HE staining, ×200). **(A)** Control group: normal epidermal and dermal structure. **(B)** Low-dose DNCB group (0.1%): epidermal thickening (red arrow), incomplete keratinization (yellow arrow), mild hemorrhage (blue arrow), inflammatory cell infiltration. **(C)** High-dose DNCB group (0.5%): marked epidermal thickening (red arrow), hyperkeratosis (yellow arrow), necrotic cell debris (black arrow), more prominent inflammatory infiltration (blue arrow).

To characterize the dermatopathological changes induced by DNCB in the AD mouse model, we performed hematoxylin and eosin (H&E) staining on dorsal skin tissues. Control mice exhibited intact and normal epidermal and dermal structures without evident pathological abnormalities (**Figure 1A**). Compared to controls, the low-dose DNCB (0.1%) group displayed pronounced epidermal thickening (red arrows), focal parakeratosis (yellow arrows), mild hemorrhage (blue arrows), and moderate inflammatory infiltration (**Figure 1B**). Pathological changes in the high-dose DNCB (0.5%) group were markedly more severe, featuring substantial epidermal thickening (red arrows), hyperkeratosis (yellow arrows), conspicuous necrotic cellular debris (black arrows), and extensive inflammatory cell infiltration (blue arrows) (**Figure 1C**).

**Figure 2:**
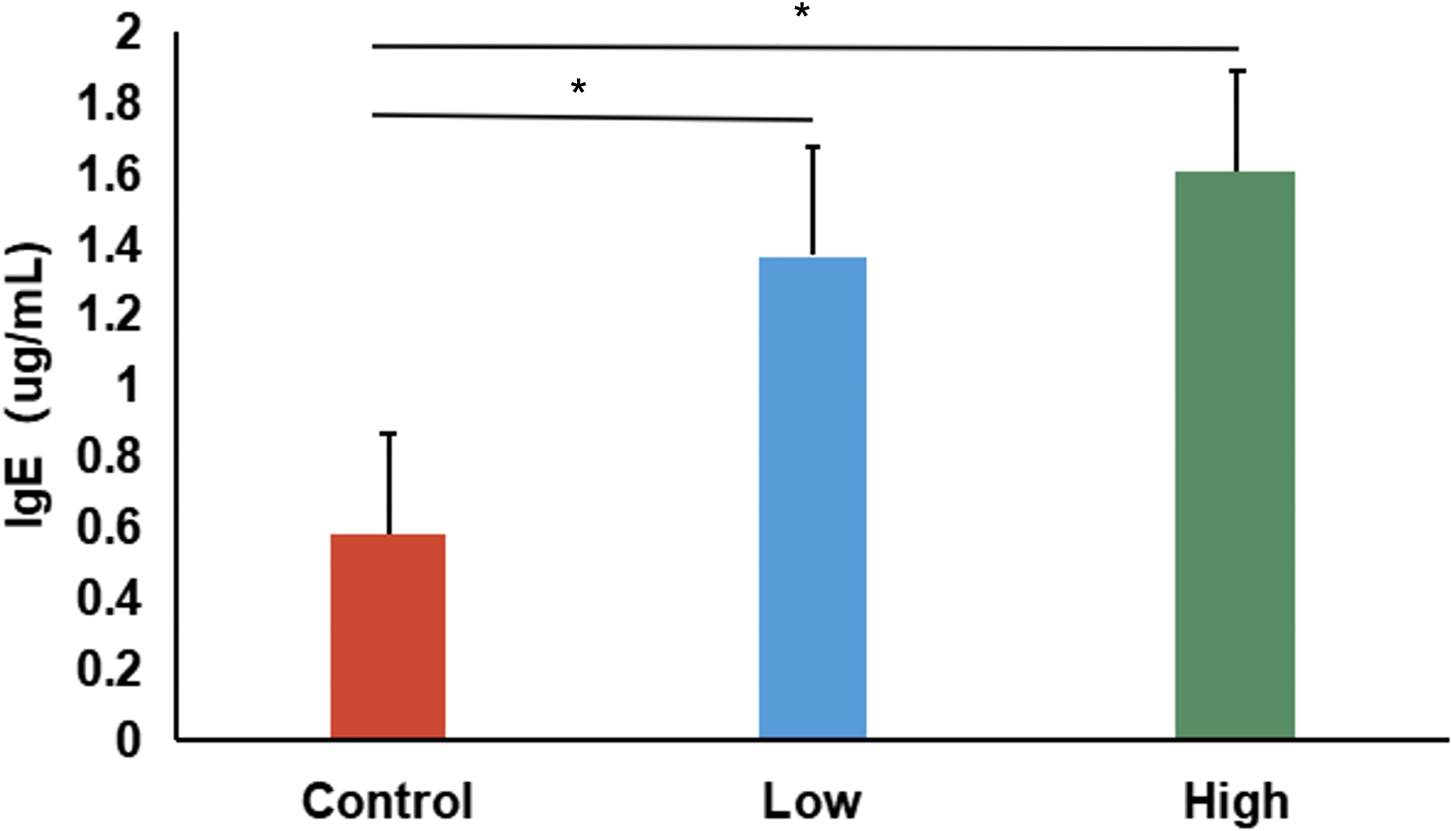
Serum IgE levels in control, low-dose, and high-dose DNCB-induced mice. Values are presented as mean ± SD (n=6 per group). Significant increases in serum IgE levels were observed in both DNCB-treated groups compared with the control group, with a clear dose-dependent pattern. (*P<0.05 vs. control group).

To further confirm the effectiveness of the DNCB-induced AD model, serum IgE concentrations were measured. Both low- and high-dose DNCB groups exhibited significantly elevated serum IgE levels compared to controls (P < 0.05, **Figure 2**), displaying a clear dose-dependent relationship. This finding, along with the dermatopathological alterations (epidermal thickening, inflammatory infiltration, abnormal keratinization, and necrosis), validated the successful establishment of an AD mouse model.

#### 3.1.2 Gut microbial diversity analysis

**Figure 3:**
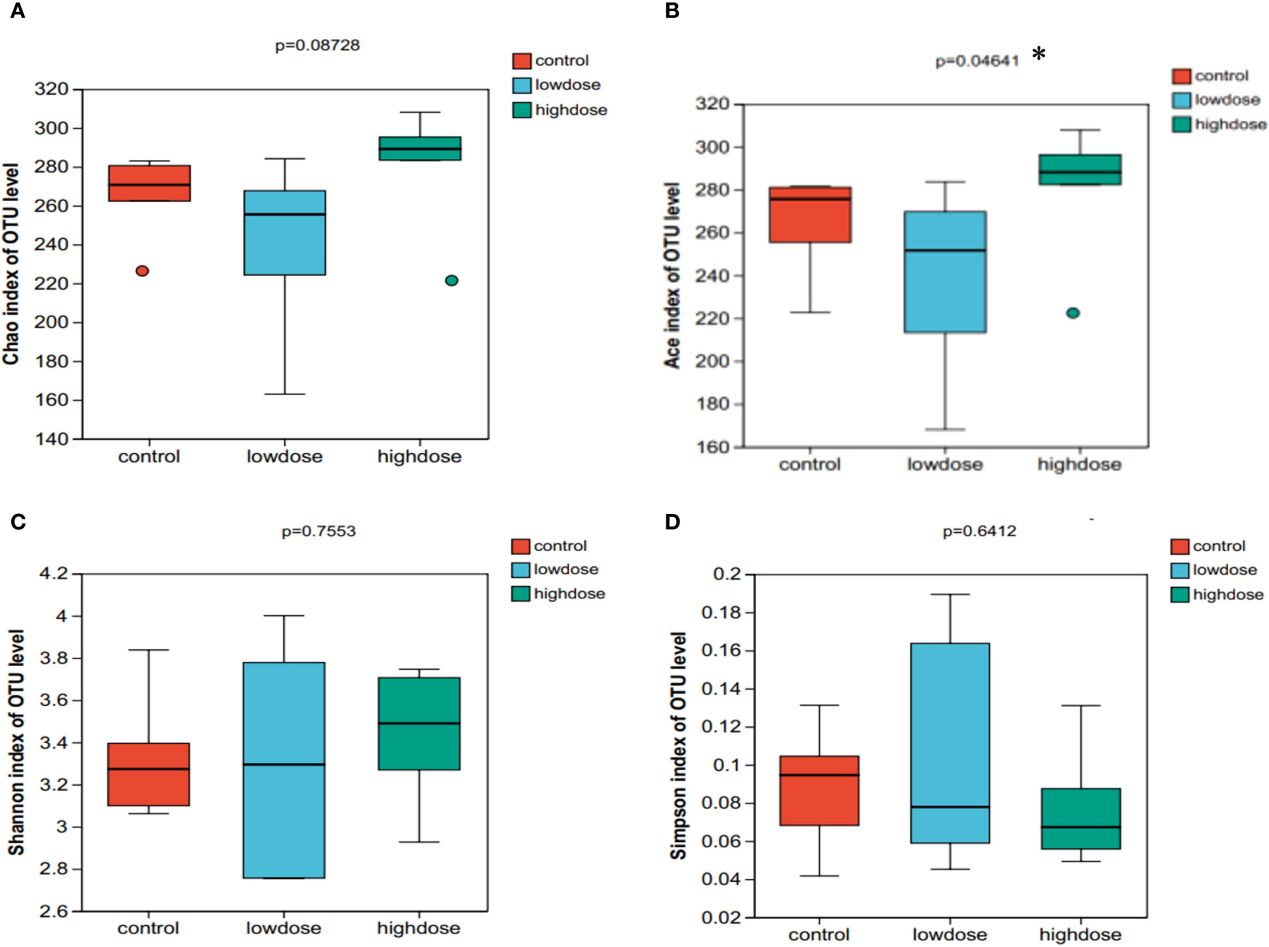
Alpha diversity indices of gut microbiota in control, low-dose, and high-dose DNCB-induced mice. **(A)** Chao index, **(B)** Ace index, **(C)** Shannon index, and **(D)** Simpson index. Statistical differences were evaluated using the Kruskal-Wallis H test; P values are indicated above each panel. (*P < 0.05).

We evaluated gut microbial alpha diversity in mice by measuring the Chao, ACE, Shannon, and Simpson indices (**Figure 3**). Compared to controls, mice in the low-dose DNCB group showed a trend toward reduced diversity. However, the differences were not statistically significant (P > 0.05). Notably, the ACE index was significantly higher in the high-dose group compared to both low-dose and control groups (P < 0.05, **Figure 3B**), indicating dose-dependent impacts on specific microbial diversity metrics. However, other indices (Chao, Shannon, Simpson) showed no significant differences across groups (**Figures 3A, C, D**). Thus, DNCB exposure appeared to influence gut microbiota alpha diversity in a dose-related manner.

#### 3.1.3 Gut microbiota composition and biomarker analysis

**Figure 4:**
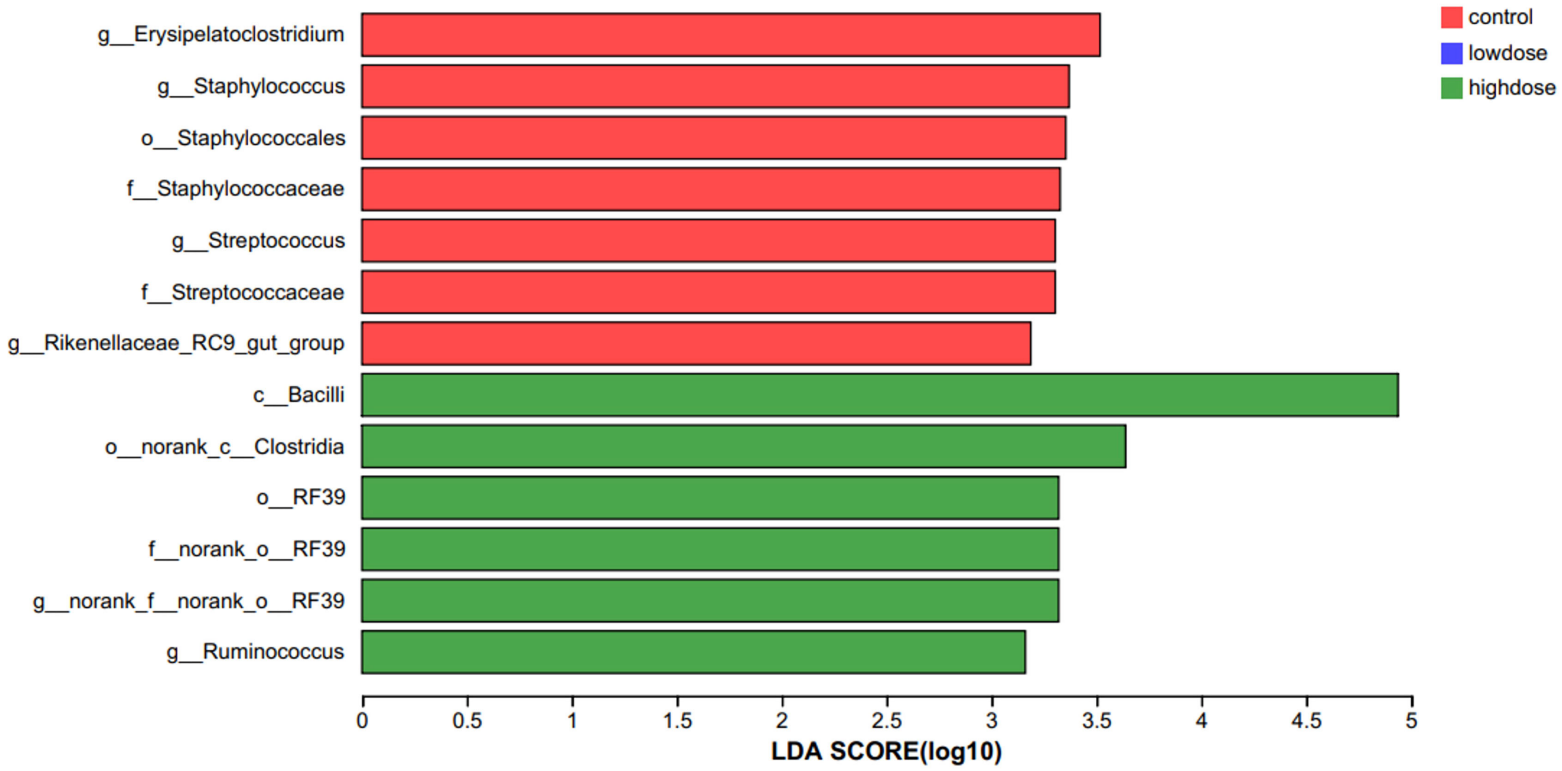
LEfSe analysis identifying characteristic bacterial taxa among experimental groups. Linear discriminant analysis Effect Size (LEfSe) identified bacterial taxa significantly enriched in each group (LDA > 2, P<0.05). The high-dose DNCB-treated group was characterized by enrichment of Bacilli, Clostridia, and Ruminococcus, whereas the control group showed enrichment of Erysipelatoclostridium, Staphylococcus, Streptococcus, and Rikenellaceae_RC9_gut_group.

Gut microbiota profiling at the phylum level revealed Bacteroidota and Firmicutes as predominant taxa, with their proportions significantly altered by DNCB exposure (**Table 2**). Using Linear discriminant analysis Effect Size (LEfSe), we further identified taxa significantly associated with each group. The high-dose DNCB group exhibited enriched populations of Bacilli, Clostridia, and Ruminococcus. In contrast, the control group was characterized by Erysipelatoclostridium, Staphylococcus, Streptococcus, and Rikenellaceae_RC9_gut_group (**Figure 4**). These findings underscored the profound restructuring of gut microbial composition following DNCB exposure, potentially related to AD progression.

**Table 2:** Top three most abundant gut microbial taxa in each group.

Group	Phylum	Class	Order	Family	Genus
Control	*Bacteroidota* (73.50%)	*Bacteroidia* (73.50%)	*Bacteroidales* (73.50%)	*Muribaculaceae* (54.02%)	*norank_f:Muribaculaceae* (52.50%)
	*Firmicutes* (23.24%)	*Bacilli* (15.10%)	*Lactobacillales* (12.21%)	*Prevotellaceae* (16.28%)	*Lactobacillus* (12.09%)
	*Verrucomicrobiota* (1.62%)	*Clostridia* (8.13%)	*Lachnospirales* (4.98%)	*Lactobacillaceae* (12.09%)	*Prevotellaceae_UCG-001* (12.91%)
Low-dose	*Bacteroidota* (78.15%)	*Bacteroidia* (78.15%)	*Bacteroidales* (78.14%)	*Muribaculaceae* (55.07%)	*norank_f:Muribaculaceae* (53.91%)
	*Firmicutes* (13.83%)	*Bacilli* (10.97%)	*Lactobacillales* (9.51%)	*Prevotellaceae* (20.00%)	*Lactobacillus* (9.50%)
	*Verrucomicrobiota* (5.44%)	*Verrucomicrobiae* (5.44%)	*Verrucomicrobiales* (5.44%)	*Lactobacillaceae* (9.50%)	*Alloprevotella* (13.25%)
High-dose	*Bacteroidota* (68.31%)	*Bacteroidia* (68.31%)	*Bacteroidales* (68.30%)	*Muribaculaceae* (49.83%)	*norank_f:Muribaculaceae* (49.28%)
	*Firmicutes* (28.26%)	*Bacilli* (19.87%)	*Lactobacillales* (17.45%)	*Prevotellaceae* (12.22%)	*Lactobacillus* (17.28%)
	*Actinobacteriota* (1.82%)	*Clostridia* (8.38%)	*Lachnospirales* (3.44%)	*Lactobacillaceae* (17.28%)	*Prevotellaceae_UCG-001* (6.50%)

#### 3.1.4 Functional prediction of gut microbiota

We employed COG-based functional prediction to assess the impact of DNCB exposure on microbiota function (**Supplementary Figure S1**). No significant differences in functional pathways were observed among the control, low-dose, and high-dose groups (all P > 0.05; 95% CI including zero, **Supplementary Figure S1B**). Thus, DNCB exposure did not significantly alter the gut microbial functional composition at the doses used in this study, despite some trends in functional shifts.

Collectively, our animal studies demonstrated characteristic AD pathological features and significant gut microbial restructuring, particularly in the abundances of *Firmicutes* and *Bacteroidota*. These microbiota alterations correlated closely with immunological changes, laying a robust foundation for further human investigations into similar microbiota and dietary relationships. Accordingly, we avoid inferring functional consequences from the murine compositional shifts alone. Future work will employ shotgun metagenomics or metabolomics to quantify pathway activity and validate these exploratory predictions directly.

### 3.2 Human study

Building on the findings from the animal experiments regarding AD-associated gut microbiota and their correlations with host immune phenotypes, our study further validated these specific microbial features in human AD patients. It systematically analyzed the impact of dietary patterns on the gut microbiota, aiming to establish clinical evidence for the “diet–gut microbiota-AD” axis in disease pathogenesis.

#### 3.2.1 Participant characteristics and dietary structure analysis

**Figure 5:**
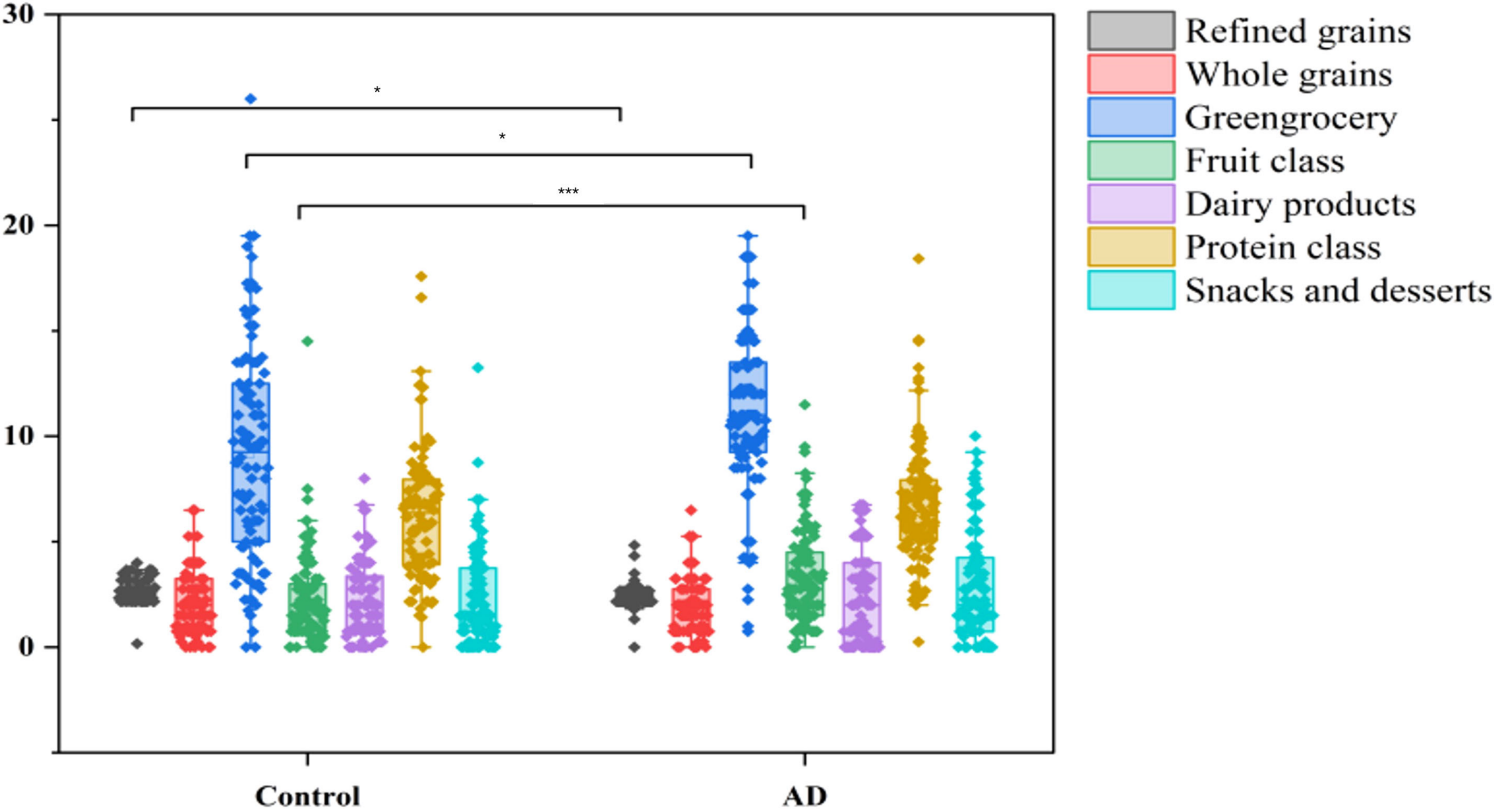
Dietary intake differences between AD patients and controls. Boxplots illustrate the intake frequency of dietary categories, showing significantly reduced consumption of refined grains (***P < 0.001) and increased intake of vegetables and fruits (*P < 0.05 and ***P < 0.001, respectively) in the AD group compared with controls.

A total of 204 participants were included in this study, including 102 AD patients and 102 healthy controls. To compare differences in dietary intake patterns between AD patients and control groups, the frequency of intake of different food groups was analyzed. The results showed that AD patients had a significantly lower intake of refined grains (P < 0.001) but a significantly higher intake of vegetables and fruits compared to the control group (P < 0.05, P < 0.001; **Figure 5**). These differences suggested that AD patients may prefer a diet rich in dietary fiber and anti-inflammatory components, which may be related to alterations in the gut microbiota and the onset and progression of AD.

**Figure 6:**
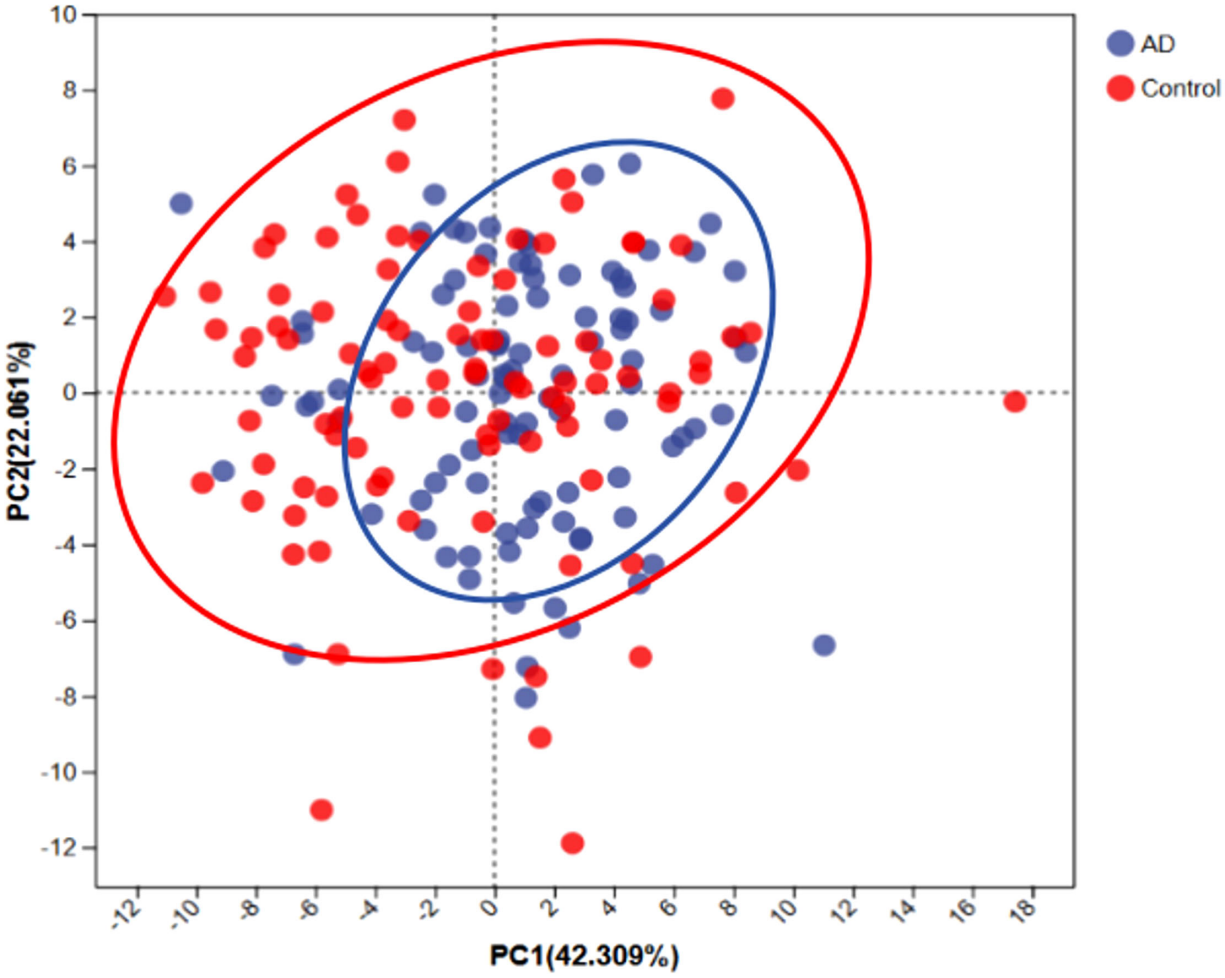
Principal component analysis (PCA) of dietary patterns between AD patients and controls. PCA scatterplot indicates distinct dietary patterns between AD patients (blue) and controls (red). Ellipses represent the 95% confidence intervals for each group, suggesting that AD patients tend toward a dietary pattern characterized by higher fiber and anti-inflammatory food intake.

To assess the differences in overall dietary patterns between the AD and control groups, we performed principal component analysis (PCA). PCA showed a clear trend of segregation between the AD and control groups (**Figure 6**), indicating significant differences in overall dietary patterns between the two groups. Combined with meal frequency analysis, AD patients exhibited a decrease in refined grain intake and an increase in vegetable and fruit consumption, suggesting that dietary patterns may be associated with changes in gut microbiota composition and the occurrence and progression of AD.

#### 3.2.2 Analysis of gut microbiota diversity and structural differences

**Figure 7:**
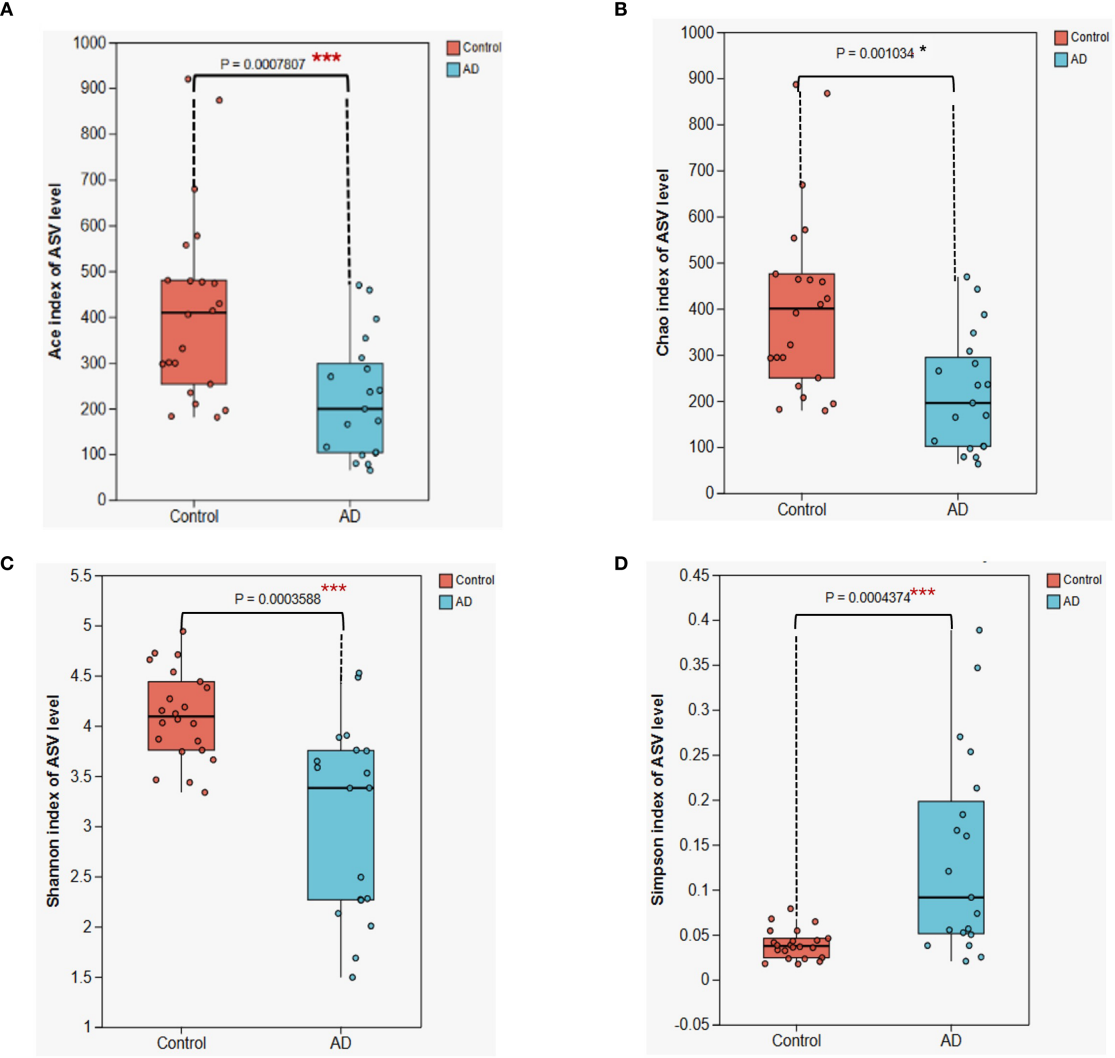
Alpha diversity analysis of gut microbiota between AD patients and control subjects. **(A)** ACE index **(B)** Chao index; **(C)** Shannon index; **(D)** Simpson index. Boxplots illustrate that all four diversity indices are significantly lower in the AD group compared with the control group (*P < 0.05, *** P < 0.001), suggesting a substantial reduction in gut microbiota diversity in AD patients. Differences were assessed using the Kruskal-Wallis H test.

To determine differences in gut microbiota diversity between AD patients and healthy controls, we assessed alpha diversity indices, including the ACE, Chao, Shannon, and Simpson indices. Results showed significant reductions in ACE (P < 0.001), Chao (P < 0.05), Shannon (P < 0.001), and Simpson (P < 0.001) indices in the AD group compared with the control group (**Figure 7**). These findings indicate notable declines in gut microbial richness and diversity among AD patients. Such pronounced dysbiosis in gut microbial ecosystems likely contributed to the onset and progression of AD.

#### 3.2.3 Microbiota composition and LEfSe marker analysis

**Figure 8:**
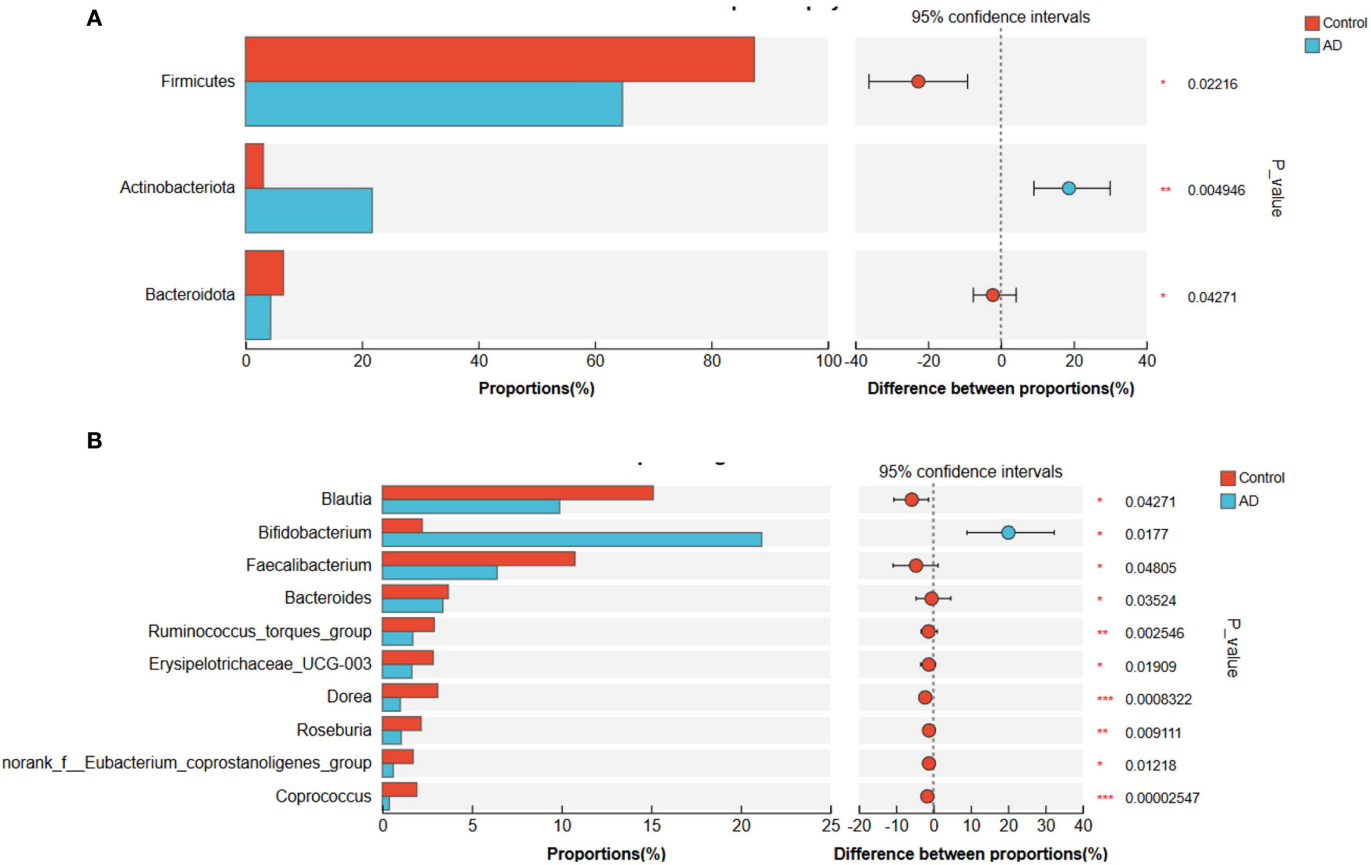
Gut microbiota composition differences at the phylum and genus levels between AD patients and controls. **(A)** Phylum-level differences showing significantly reduced Firmicutes and Bacteroidota, and significantly increased Actinobacteriota in the AD group compared to controls. **(B)** Genus-level differences showing significantly increased Bifidobacterium and significantly decreased Blautia in the AD group. Faecalibacterium showed a decreasing trend in the AD group but did not reach statistical significance (P>0.05). Differences were evaluated with a two-sided proportion test. (*P<0.05, **P<0.01, ***P<0.001).

To further examine differences in gut microbiota composition between the AD and control groups, we conducted analyses at the phylum and genus levels (**Figure 8**). At the phylum level, the relative abundances of *Firmicutes* (P < 0.05) and *Bacteroidota* (P < 0.05) were significantly lower in AD patients, whereas Actinobacteriota showed a significant increase (P < 0.01; **Figure 8A**). At the genus level, Bifidobacterium was significantly enriched in the AD group (P < 0.05), whereas Blautia was significantly reduced (P < 0.05). Fecalibacterium displayed a downward trend, though not statistically significant (P > 0.05; **Figure 8B**). These pronounced microbial composition shifts might have been closely related to gut microbial dysbiosis and AD progression.

**Figure 9:**
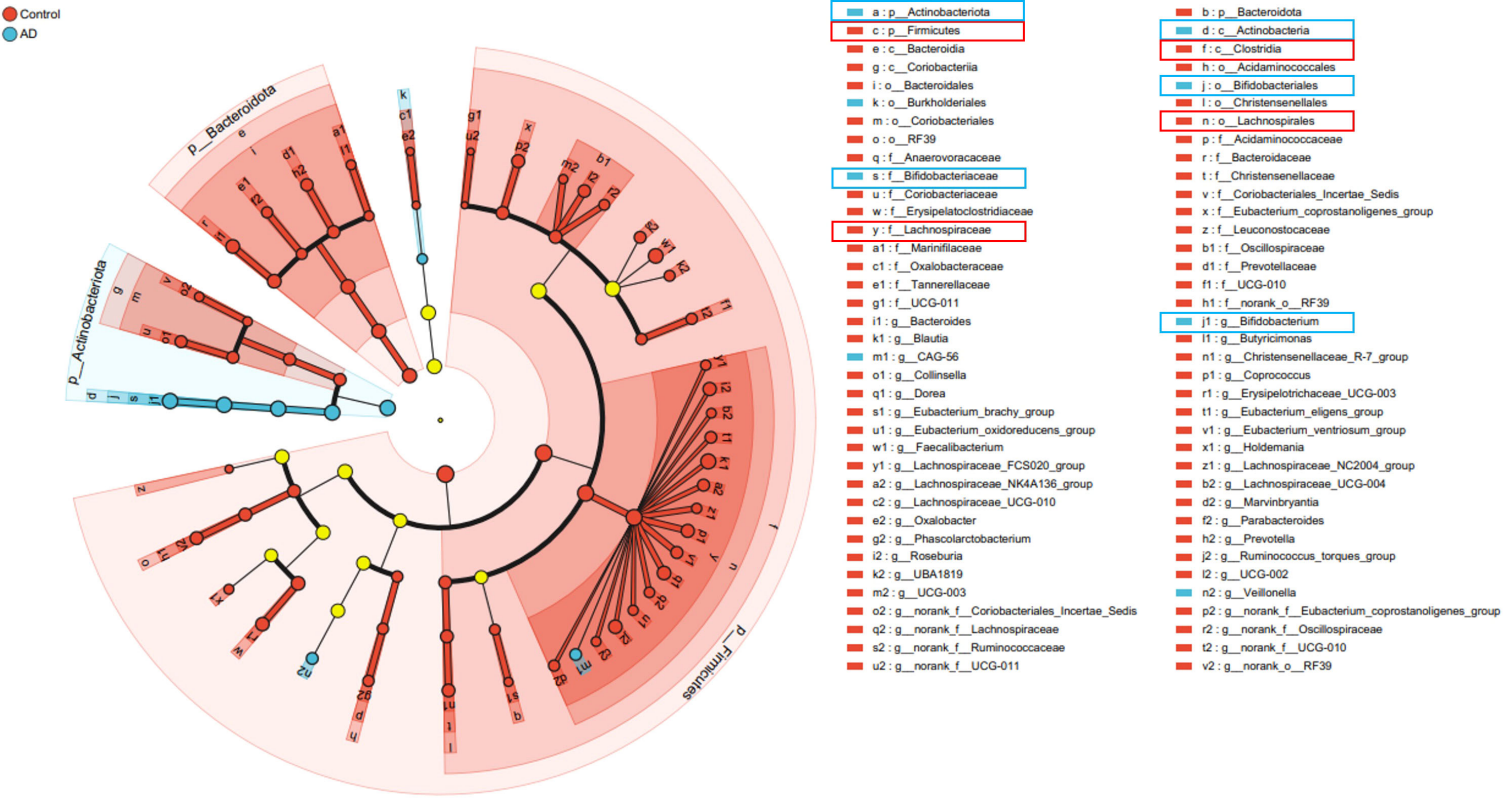
Cladogram illustrating significant differences in gut microbiota taxa between AD patients and controls identified by LEfSe analysis. Taxa significantly enriched in the AD group are marked in blue (notably Actinobacteriota and Bifidobacterium), whereas taxa enriched in the control group are marked in red (notably Firmicutes and Lachnospiraceae). Yellow circles represent taxa without significant differences between groups.

To further clarify characteristic gut microbiota changes in AD patients, we applied linear discriminant analysis effect size (LEfSe). Results revealed significant enrichment of Actinobacteriota and Bifidobacterium in AD patients, while Firmicutes and Lachnospiraceae were markedly enriched in controls (**Figure 9**). These structural microbial shifts further illustrated pronounced gut dysbiosis in AD patients, potentially influencing AD onset and development.

#### 3.2.4 Correlation analysis between gut microbiota and dietary factors

**Figure 10:**
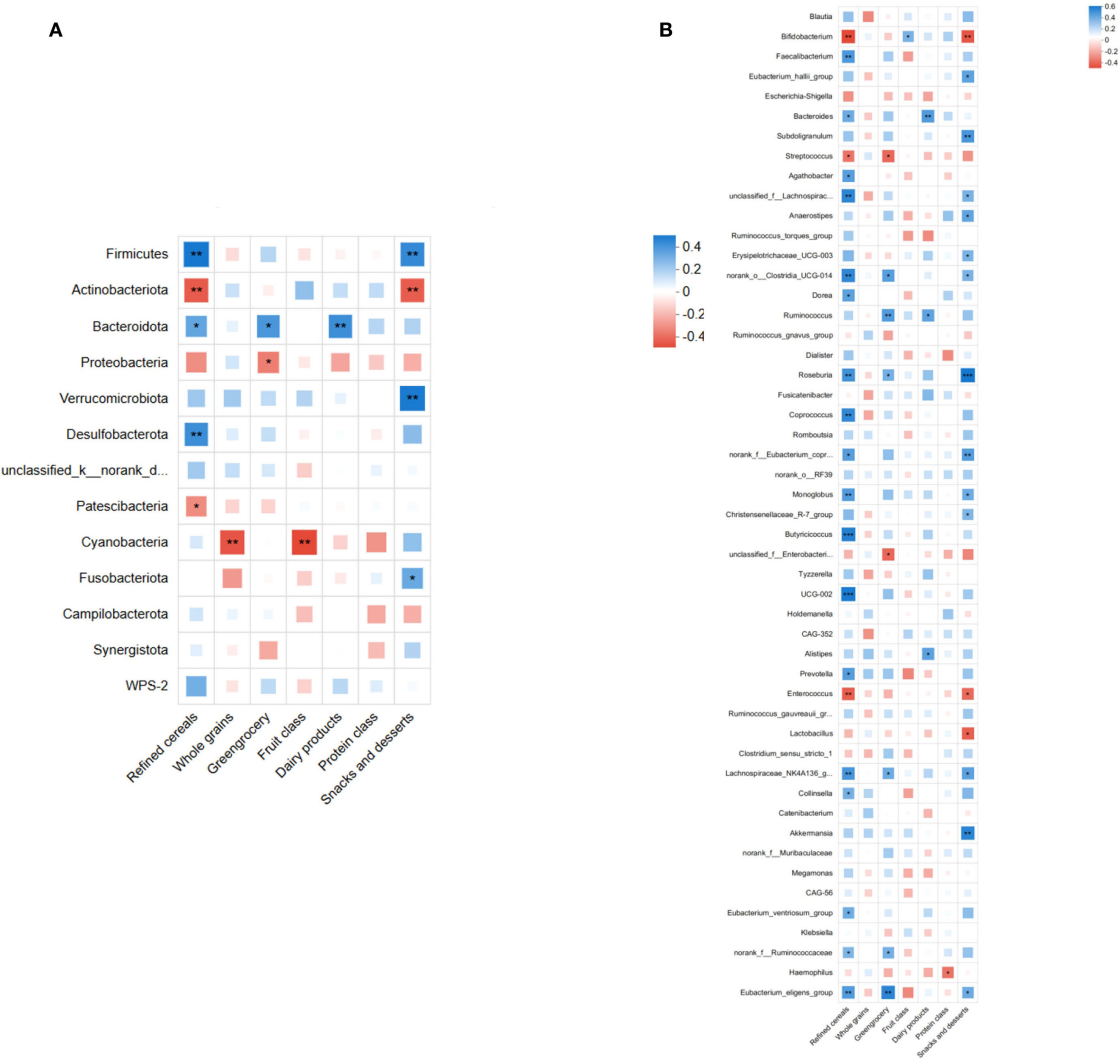
Spearman's correlation analysis between gut microbiota (top 50 taxa in relative abundance) and dietary factors. **(A)** Correlation at the phylum level, showing significant positive correlations of Firmicutes and significant negative correlations of Actinobacteriota with refined grains and snacks/desserts intake (P<0.05). **(B)** Correlation at the genus level, indicating significant positive correlations of Ruminococcus and Roseburia abundances with refined grains and vegetables intake (P <0.05). Roseburia abundance also correlated positively with snacks and desserts (P<0.05). Lachnospiraceae abundance correlated positively with refined grains and vegetables intake (P<0.05), showing a non-significant positive correlation trend with snacks and desserts (P> 0.05). (*P<0.05, **P<0.01, ***P<0.001; blue indicates positive correlation, red indicates negative correlation).

To explore relationships between gut microbiota structure and dietary factors, we performed Spearman correlation analyses comparing the top 50 microbial taxa (phylum and genus levels) with different dietary categories. At the phylum level, Firmicutes showed a significant positive correlation with refined grains and snacks/sweets intake (P < 0.05), while Actinobacteriota displayed a significant negative correlation with these dietary categories (P < 0.05; **Figure 10A**). At the genus level, Ruminococcus and Roseburia correlated positively with refined grains and vegetables intake (P < 0.05); Roseburia also positively correlated with snacks/sweets intake (P < 0.05). Lachnospiraceae correlated significantly positively with refined grains and vegetables (P < 0.05) and showed a non-significant positive trend with snacks/sweets (P > 0.05; **Figure 10B**). These results implied that dietary factors might influence AD pathogenesis by modulating gut microbial composition.

**Figure 11:**
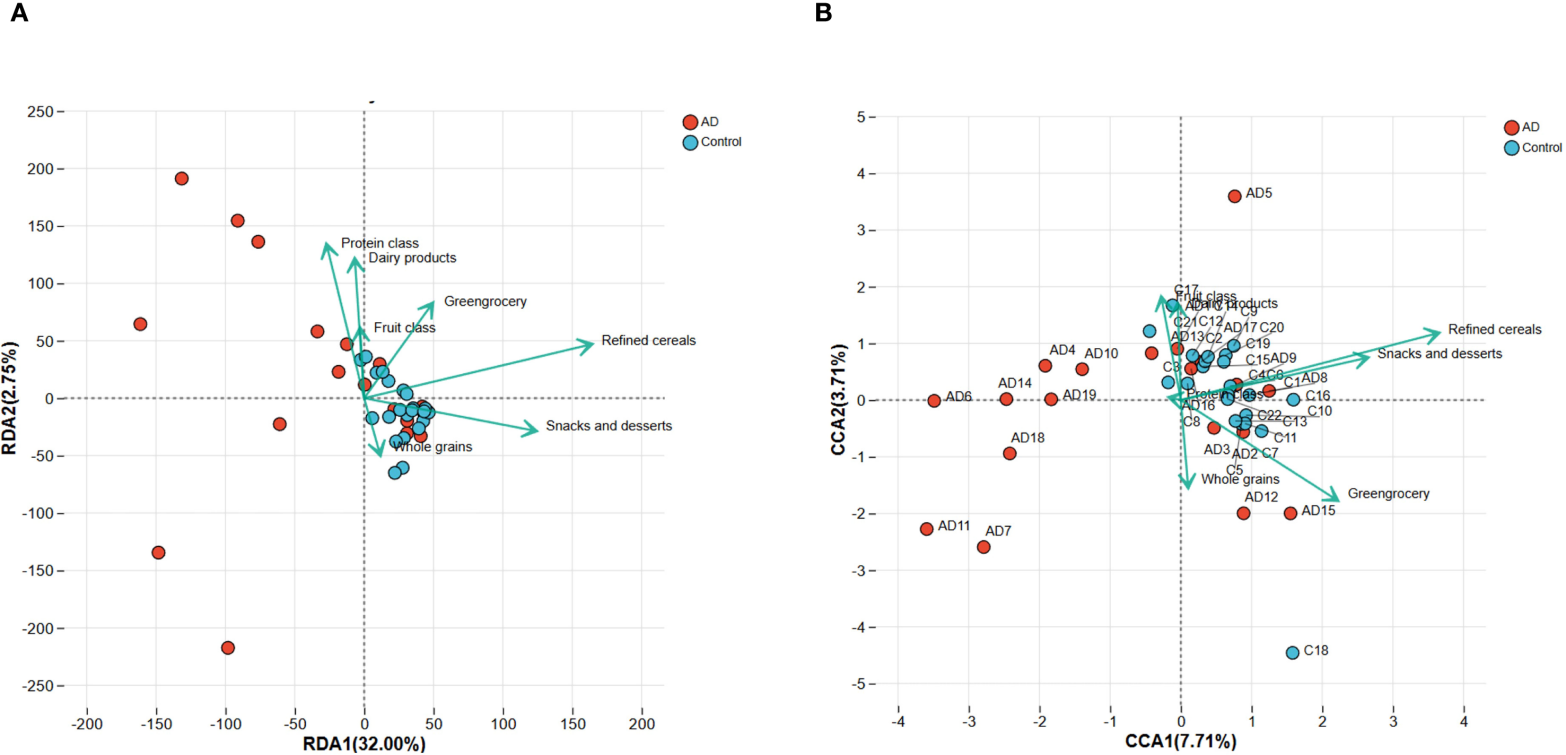
RDA and CCA analyses demonstrating the associations between dietary factors and gut microbiota composition in AD patients and controls. **(A)** RDA plot at the phylum level indicates clear separation of microbiota composition between AD and control groups. Dietary factors, including refined cereals, greengrocery, and snacks and desserts, significantly correlated with microbiota variation. **(B)** CCA plot at the genus level similarly highlights significant differences in microbiota structure between groups, further confirming the influence of dietary factors. Arrows indicate the direction and magnitude of the correlations.

To further establish associations between dietary factors and gut microbiota structure, redundancy analysis (RDA) and canonical correspondence analysis (CCA) were conducted at the phylum and genus levels, respectively. RDA (phylum level) revealed clear distinctions between AD and control samples, with refined grains, vegetables, and snacks/sweets having a substantial impact on microbiota structure (**Figure 11A**). Similarly, CCA (at the genus level) confirmed a distinct separation between the AD and control groups, highlighting the significant impact of dietary factors on genus composition (**Figure 11B**). Collectively, these results support that dietary factors likely influence AD development by shaping gut microbiota structure.

#### 3.2.5 Functional prediction of gut microbiota

**Figure 12:**
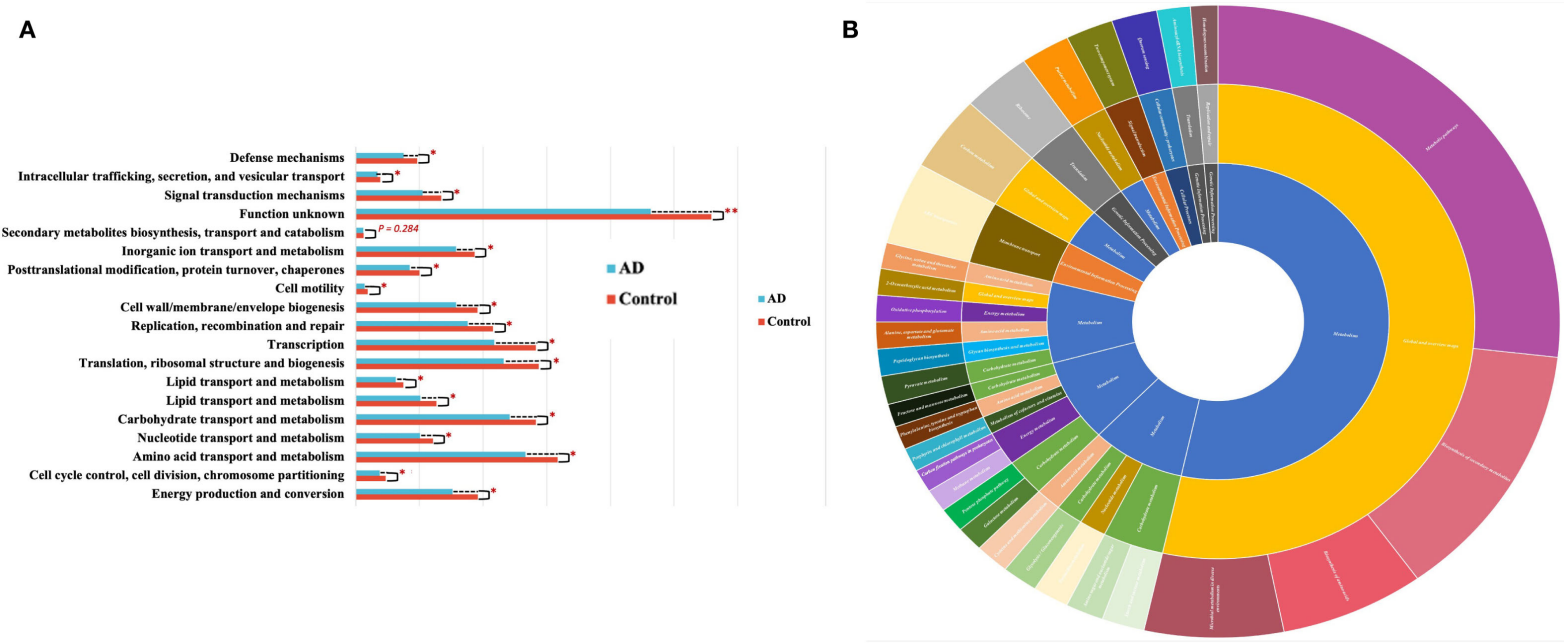
Functional prediction of gut microbiota based on COG and KEGG analyses. **(A)** COG functional category comparisons between AD patients and controls, showing significantly decreased activities in carbohydrate metabolism, amino acid metabolism, and energy metabolism pathways in the AD group (*P<0.05). **(B)** KEGG functional annotation depicted by hierarchical sunburst chart (Level 1-Level 3), highlighting the major functional categories and emphasizing the importance of metabolic pathways such as carbohydrate, amino acid, and energy metabolism within gut microbiota.

To further explore functional profiles of gut microbiota in AD patients, we performed functional predictions using the COG and KEGG databases. COG analysis demonstrated significant reductions in carbohydrate transport and metabolism, amino acid transport and metabolism, and energy production and conversion pathways in AD patients compared with controls (P < 0.05; **Figure 12A**). Additionally, KEGG functional annotation (**Figure 12B**) further highlighted the importance of these metabolic pathways within overall gut microbiota function. These results collectively suggested significant functional metabolic dysbiosis within the gut microbiota of AD patients, potentially involved in disease pathophysiology.

The findings from this human investigation substantially confirmed and extended our animal model data, clearly showing reduced gut microbiota diversity, significant compositional restructuring, and marked microbial functional disturbances in AD patients. Notably, these alterations were closely linked to dietary patterns. Overall, this study highlighted the critical role that dietary-gut microbiota interactions play in AD pathogenesis, providing robust clinical evidence to support future strategies in dietary intervention and gut microbiota modulation for AD prevention and treatment.

## 4 Discussion

In this study, animal experiments combined with human population surveys comprehensively explored the relationships between dietary factors, gut microbiota, and atopic dermatitis (AD), highlighting the potential mechanisms through which gut dysbiosis may contribute to AD development.

Results from the animal experiments demonstrated that DNCB successfully induced a mouse model exhibiting typical AD features, including significant pathological skin changes (epidermal thickening, inflammatory infiltration, hyperkeratosis, and necrotic cell debris) and dose-dependent increases in serum IgE levels. These findings are consistent with previous studies, as elevated IgE is widely recognized as a key immunological hallmark of AD (21). Simultaneously, significant alterations were observed in gut microbial diversity and composition in these mice, particularly with notable changes in the proportions of Firmicutes and Bacteroidota. Furthermore, specific microbial taxa, such as Bacilli, Clostridia, and Ruminococcus, were notably enriched in the high-dose group, aligning well with existing theories that propose gut dysbiosis may exacerbate inflammatory immune responses (22, 23).

In the human population study, we validated and expanded the findings from the animal model. AD patients exhibited significantly reduced gut microbiota diversity, consistent with previous research indicating that decreased microbial diversity often correlates with immune dysfunction and increased inflammation (24). Moreover, significant compositional changes in gut microbiota were observed at both phylum and genus levels among AD patients, characterized by decreased abundances of Firmicutes and Bacteroidota and increased abundances of Actinobacteriota and Bifidobacterium, with a notable decrease in Blautia. These results suggested that microbial restructuring might directly contribute to AD pathogenesis, particularly the increased abundance of Bifidobacterium, which could correlate with immune dysregulation and enhanced inflammatory responses in patients.

Interestingly, we observed an increased abundance of *Bifidobacterium* in AD patients, which appeared counterintuitive given its traditional role as a beneficial genus associated with improved gut health. However, recent studies suggest that the effects of Bifidobacterium may be highly strain-specific and context-dependent. Certain strains may exert pro-inflammatory activity under dysregulated immune conditions, while others are protective. Moreover, host-related factors such as genetic background, immune status, and dietary environment may influence whether Bifidobacterium acts beneficially or potentially contributes to disease progression. Thus, the observed enrichment in AD patients may not necessarily contradict its probiotic reputation but instead highlight the complex host–microbiota interactions that warrant further mechanistic investigation. Given the pronounced strain heterogeneity within Bifidobacterium, our interpretation is hypothesis-generating. Definitive confirmation of functional relevance will require strain-resolved shotgun metagenomics (e.g., read- or MAG-level profiling), complementary metatranscriptomics/metabolomics, and culture-based isolation followed by functional assays and/or gnotobiotic transfer experiments.

Importantly, this study systematically investigated, for the first time, the association between dietary patterns and gut microbiota composition in AD patients. Our findings revealed that the dietary habits of AD patients deviated significantly from those of healthy controls, characterized by an increased intake of fiber-rich and anti-inflammatory foods, such as vegetables and fruits, coupled with a reduced consumption of refined grains. This dietary shift could reflect either proactive adjustments made by patients to alleviate symptoms or passive changes in dietary preferences. Another possible explanation for the observed higher intake of fruits and vegetables and lower intake of refined grains among AD patients was reverse causality. Specifically, patients may have consciously adjusted their dietary habits after the onset of the disease in an attempt to alleviate symptoms. This behavioral change could introduce bias, as the observed dietary patterns may not necessarily represent pre-disease exposures but rather reflect secondary modifications driven by health awareness. Moreover, given that our FFQ relied on retrospective self-reporting, recall bias could not be ruled out, further limiting the causal interpretation of these findings. Future longitudinal or prospective dietary assessments are needed to disentangle whether such dietary patterns are a cause or consequence of AD. Further analysis through Spearman correlation and RDA/CCA confirmed close associations between specific dietary factors (refined grains, vegetables, and fruits) and microbial taxa (such as Firmicutes, Bifidobacterium, Ruminococcus, and Roseburia). These findings align with previous studies that highlight diet as a critical modulator of gut microbial composition (25, 26). Additionally, functional prediction analyses demonstrated significant disruptions in carbohydrate, amino acid, and energy metabolism pathways within the gut microbiota of AD patients. This suggests that despite AD patients adopting healthier dietary habits, metabolic dysfunction within the gut microbiota may impair the effective utilization of nutrients, further perpetuating inflammation and immune dysregulation.

Functional predictions of gut microbiota revealed markedly reduced activity in metabolic pathways involving carbohydrates, amino acids, and energy production in AD patients. Prior research indicated that abnormal gut microbial metabolism could exacerbate gut barrier dysfunction and immune imbalance, thereby promoting AD development and progression. Thus, significant alterations in microbial metabolic function further support the hypothesis of gut microbial dysbiosis as an important factor in AD pathophysiology.

Despite providing comprehensive data support through animal and human studies, this research has certain limitations. Firstly, due to the cross-sectional design, causal relationships between dietary factors and microbiota alterations remain unclear and require confirmation through prospective or interventional studies. Secondly, the lack of significant changes in microbial functional profiles observed in animal experiments may be attributed to insufficient sample size or exposure duration. Future studies should consider longer exposure periods or increased sample sizes. Moreover, the absence of significant functional alterations despite compositional shifts may also reflect methodological limitations. For instance, the 4-week exposure duration may not have been sufficient to induce measurable metabolic reprogramming, and the sequencing depth and sample size could have reduced the sensitivity to detect subtle changes. This study’s functional predictions of microbial communities were performed using PICRUSt2, as described in the Methods section. However, PICRUSt2 relies on reference genomes and predictive algorithms rather than direct metagenomic sequencing, which might have limited the accuracy of functional inferences. This methodological limitation may partly explain the absence of significant functional alterations in the animal experiments despite compositional changes. Despite compositional restructuring, the absence of significant functional changes in mice underscores the limitations of inference-based tools such as PICRUSt2 and the relatively short exposure and sequencing depth. Therefore, these functional outputs are preliminary, best viewed as hypothesis-generating signals rather than definitive evidence of pathway perturbation. To overcome this limitation, future studies should consider integrating direct metagenomic or metabolomic profiling, alongside extended exposure periods and larger cohorts, to more accurately evaluate the functional consequences of gut microbiota alterations. Additionally, although our FFQ was adapted from the validated CHNS instrument, the shortened version used in this study has not been independently validated. This may introduce recall bias and dietary misclassification, which could influence the observed associations between diet and microbiota. Therefore, the results should be interpreted cautiously, and future studies are encouraged to use fully validated FFQs or multiple 24-hour dietary recalls to improve dietary assessment. Furthermore, the FFQ in this study assessed dietary intake over the past 12 months, whereas stool samples for microbiota analysis were collected simultaneously. This temporal mismatch may weaken the precision of observed diet–microbiota associations. Future research should incorporate repeated dietary assessments alongside longitudinal microbiota sampling to more accurately capture dynamic diet–microbiota interactions. Another limitation is that our FFQ did not separately categorize probiotic-containing foods and beverages (e.g., yogurt, cultured drinks). Although such items were grouped within the general dairy category, this decision likely biased our results by underestimating or obscuring the specific contributions of probiotic foods to gut microbiota composition and AD pathophysiology. Given probiotic foods’ strong and well-documented effects on microbial diversity and immune regulation, future research should incorporate dietary assessments that specifically and independently capture probiotic intake to more accurately evaluate their role in modulating the diet-microbiota-AD axis. We did not adjust for potential confounders such as BMI, medication use, socioeconomic status, and allergic comorbidities. Furthermore, our study did not systematically collect detailed clinical information such as disease severity scores (e.g., SCORAD, EASI), disease duration, comorbid allergic conditions (asthma, allergic rhinitis, food allergy), prior treatment history, anthropometric parameters (BMI, weight, height), perinatal and early-life factors (birth mode, feeding type), and lifestyle/environmental factors (urban versus rural origin, exercise habits). The absence of these data limited our ability to perform stratified analyses or adjust for potentially essential modifiers of gut microbiota composition. We acknowledge this as a significant limitation, and emphasize that future studies should incorporate standardized clinimetric scales and comprehensive demographic, clinical, and lifestyle data to strengthen the reliability and interpretability of host–microbiota associations in AD. Finally, detailed sequencing statistics, such as raw read counts and post-filtering valid reads, were not archived and cannot be reported in this manuscript. This represents a substantial methodological weakness, as rarefaction curves, while useful, cannot fully substitute for actual sequencing depth metrics in ensuring reproducibility. Nevertheless, sequencing reliability was evaluated using rarefaction curves based on Shannon and Simpson indices, which reached saturation across all groups. This indicates that sequencing coverage was sufficient to capture the majority of microbial diversity present in the samples, supporting the robustness of subsequent compositional and functional analyses. However, the absence of archived raw read data may limit reproducibility; therefore, future studies should retain complete sequencing metrics to enhance methodological transparency further.

In summary, despite certain methodological limitations, including the cross-sectional design, limited collection of clinical covariates, and reliance on predictive functional inference, our study underscores the integration of animal experiments and human population data to elucidate the interplay between diet, gut microbiota, and AD pathogenesis. By demonstrating that dietary shifts and microbial dysbiosis converge on impaired microbial metabolic pathways, our work provided novel insights into how nutritional and microbial factors jointly shape AD onset and progression. These findings extend existing knowledge and offer a conceptual framework for dietary interventions and microbiota-targeted therapies. Future longitudinal and multi-omics studies with more comprehensive clinical characterization will be essential to validate and expand our observations. However, the present study already provides an important empirical basis and a forward-looking direction for the field.

## 5 Conclusion

This research has systematically examined the association between dietary factors, gut microbiota, and atopic dermatitis (AD) through a combined approach of animal experiments and human population studies. The DNCB-induced mouse model has successfully exhibited classical AD pathological characteristics and immune responses, accompanied by decreased gut microbial diversity and structural remodeling of specific microbial populations. Human studies have further confirmed significantly reduced gut microbial diversity in AD patients, characterized by decreased abundances of key taxa such as *Firmicutes* and *Bacteroidota*, and increased abundances of Actinobacteriota and Bifidobacterium. Analysis of dietary patterns has revealed a preference among AD patients for fiber-rich and anti-inflammatory foods, which is closely linked to observed shifts in microbiota. Furthermore, gut microbiota functional prediction has indicated notable dysfunction in carbohydrate, amino acid, and energy metabolism in AD patients. These findings generate hypotheses regarding diet–microbiota–AD relationships; definitive functional and causal inferences will require longitudinal designs and multi-omics validation. However, given the observational design, the results should be interpreted as hypothesis-generating, and further prospective and mechanistic studies are needed to confirm causality and inform dietary or microbiota-targeted interventions in AD management.

## Data Availability

The data supporting the findings of this study are available from the corresponding author upon reasonable request.
