# Supplementary figures and images for: Dietary modulation of gut microbiota and its role in atopic dermatitis: integrative evidence from animal and human studies
Source: Front Immunol. 2025 Oct 9;16:1635262. doi: 10.3389/fimmu.2025.1635262 (PMC12545045; doi:10.3389/fimmu.2025.1635262)

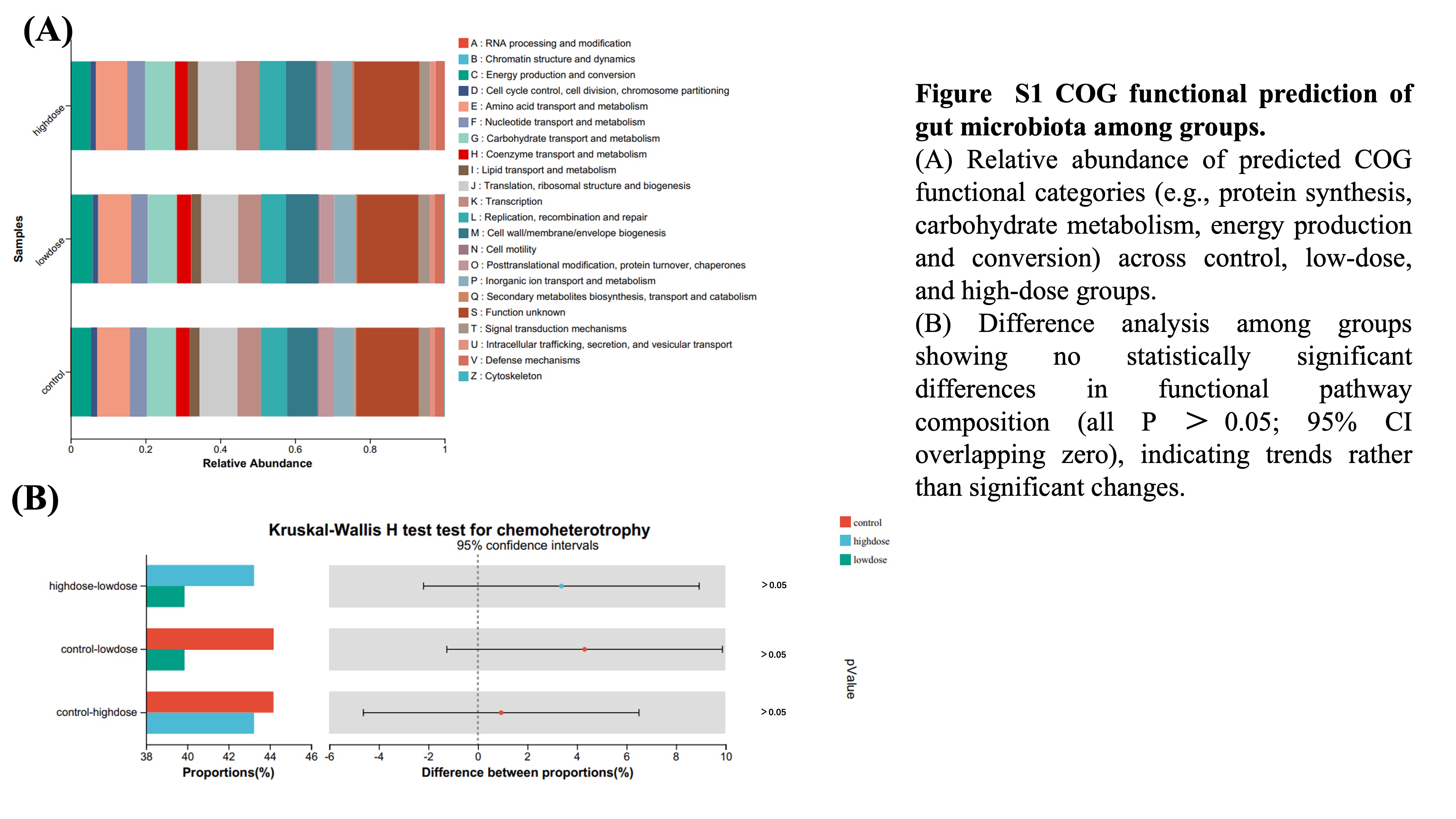

Supplement: Supplementary file 1 [file Image1.jpeg]
